# Analysis of the nucleocytoplasmic shuttling RNA-binding protein HNRNPU using optimized HITS-CLIP method

**DOI:** 10.1371/journal.pone.0231450

**Published:** 2020-04-17

**Authors:** Masato Yugami, Hideyuki Okano, Atsushi Nakanishi, Masato Yano

**Affiliations:** 1 Takeda Pharmaceutical Company, Ltd, Osaka, Japan; 2 Department of Physiology, School of Medicine, Keio University, Minato, Japan; 3 Division of Neurobiology and Anatomy, Graduate School of Medical and Dental Sciences, Niigata University, Niigata, Japan; John Curtin School of Medical Research, AUSTRALIA

## Abstract

RNA-binding proteins (RBPs) control many types of post-transcriptional regulation, including mRNA splicing, mRNA stability, and translational efficiency, by directly binding to their target RNAs and their mutation and dysfunction are often associated with several human neurological diseases and tumorigenesis. Crosslinking immunoprecipitation (CLIP), coupled with high-throughput sequencing (HITS-CLIP), is a powerful technique for investigating the molecular mechanisms underlying disease pathogenesis by comprehensive identification of RBP target sequences at the transcriptome level. However, HITS-CLIP protocol is still required for some optimization due to experimental complication, low efficiency and time-consuming, whose library has to be generated from very small amounts of RNAs. Here we improved a more efficient, rapid, and reproducible CLIP method by optimizing BrdU-CLIP. Our protocol produced a 10-fold greater yield of pre-amplified CLIP library, which resulted in a low duplicate rate of CLIP-tag reads because the number of PCR cycles required for library amplification was reduced. Variance of the yields was also reduced, and the experimental period was shortened by 2 days. Using this, we validated *IL-6* expression by a nuclear RBP, HNRNPU, which directly binds the 3’-UTR of *IL-6* mRNA in HeLa cells. Importantly, this interaction was only observed in the cytoplasmic fraction, suggesting a role of cytoplasmic HNRNPU in mRNA stability control. This optimized method enables us to accurately identify target genes and provides a snapshot of the protein-RNA interactions of nucleocytoplasmic shuttling RBPs.

## Introduction

RNA-binding proteins (RBPs) play central roles in the regulation of multiple post-transcriptional processes such as alternative splicing, mRNA stability, translation, and mRNA transport [1]. In addition, they are major components of the subcellular architecture, mediating protein-RNA interactions through translocation from the nucleus to the cytoplasm[2]. HITS-CLIP (a.k.a. CLIP-seq.), UV crosslinking immunoprecipitation (CLIP) combined with high-throughput sequencing (HITS), is a powerful technique to identify the RNA binding sites for any RBPs at the transcriptome wide [3]. HITS-CLIP also has become an indispensable tool for the investigation of molecular mechanisms and biologic roles of RBPs [3–5]. In the original CLIP method, reverse transcription must proceed from a 3’ ligated linker to a 5’ ligated linker, bypassing a short polypeptide that remains at the UV-induced crosslinking site. In over 80% of reactions, reverse transcription stalls at the crosslinking site, resulting in a truncated cDNA lacking the 5’ linker site that is necessary to amplify adapter-attached cDNA for next-generation sequencing [6, 7]. The iCLIP method, which was developed to overcome this issue, enables PCR amplification of truncated products by circularizing and re-linearizing truncated DNAs to generate the 5’ and 3’ adapter sites after reverse transcription. Although iCLIP has been used successfully to uncover the function of many RBPs [8, 9], it is technically challenging to perform, with many steps over several days. This often leads to the loss of RNAs and cDNAs due to manipulation of the very small quantities of RNA interacting with an RBP. To overcome these issues, derivative iCLIP methods such as BrdU-CLIP, FAST-iCLIP, eCLIP, and irCLIP were developed [10–14]. However, these methods remain complicated, time-consuming, and difficult to perform due to the lack of a technical positive control. Another limitation of CLIP is that it can only be used to explore protein-RNA interactions in a specific subcellular compartment, such as several types of RNA granules, also known as non-membrane organelle. Therefore, further optimization of CLIP would be beneficial.

HNRNPU was originally identified as a component of heterogeneous ribonucleoprotein (hnRNP) complexes and is also known as nuclear scaffold attachment factor A (SAF-A) [15, 16]. HNRNPU has a DNA binding domain at the N-terminus and also an RNA-binding domain known as an RGG domain at the C-terminus. This ability to bind both DNA and RNA permits HNRNPU to perform many functions, including transcriptional regulation, nuclear matrix/scaffold attachment [17–20], and alternative splicing [21–23]. Although HNRNPU has been reported to stabilize the mRNAs of insulin and the inflammatory cytokines IL-6 and IL-1β by binding the 3’-UTR [24–26], the precise binding sites and motifs in 3’-UTR RNA are not known. Despite accumulating evidence that HNRNPU regulates mRNA stability, direct evidence of HNRNPU binding to these mRNAs is lacking, perhaps due to insufficient sensitivity of CLIP methodology for nucleocytoplasmic shuttling RBPs.

We improved CLIP method, which is based on BrdU-CLIP and permits generation of a high-yield library, improves reproducibility, and minimizes CLIP-tag duplicates. Our method includes universal control RNAs and an unshuttling RBP (HNRNPC) to ascertain the efficacy of CLIP cDNA library generation from CLIP RNA. Functional analysis using our CLIP with HNRNPU combined with subcellular fractionation revealed previously unknown HNRNPU binding regions in the 3’-UTR of *IL-6* mRNA. Our data illustrate that our protocol is a powerful tool for investigating shuttling RBPs.

## Materials and methods

### Antibodies

The anti-HNRNPU antibody used for immunoprecipitation (IP) and immunofluorescence (IF) was purchased from Santa Cruz (CA, USA) (sc-32315), and the anti-HNRNPU antibody used for Western blotting (WB) was purchased from Bethyl (A300-690A). Anti-HNRNPC antibodies for IP and WB were purchased from Santa Cruz (sc-32308) and MBL (RN052PW), respectively. The anti-GAPDH antibody used for IF was purchased from Abcam (ab9485). The anti-Actin antibody used for WB was purchased from CHEMICON (MAB1501). The anti-Lamin B1 antibody for WB was purchased from Abcam (ab133741).

### Cell culture

HeLa cells (ATCC) were cultured at 37°C, 5% CO_2_, in DMEM (Life Technologies) containing 10% fetal bovine serum (FBS) (Gibco) and penicillin–streptomycin (Gibco).

### siRNA transfection and P/I induction

HeLa cells were seeded at a density of 1.0 x 10^4^ cells/well in 96-well cell culture plates. After a 24 hours incubation, the cells were transfected with 20 nM HNRNPU Silencer Select siRNA (s6743; Ambion) or Silencer Select Negative Control No.1 siRNA (AM4611; Ambion) using Opti-MEM I medium (Life Technologies) and Lipofectamine RNAiMax (Life Technologies), according to the manufacturer’s instructions, and then were incubated at 37 °C for 72 hours. The cells were stimulated with 50 ng/mL PMA (Sigma) and 0.5 μM calcium ionophore A23187 (Sigma) for 2, 4, or 24 hours, and subsequently subjected to TaqMan and Western blot assays.

### Quantitative RT-PCR (TaqMan) assay

Total RNAs were extracted from siRNA-transfected HeLa cells using an RNeasy 96 kit (QIAGEN). The cDNA from 100 ng of total RNA was synthesized using a High-Capacity cDNA Reverse Transcription Kit (Applied Biosystems) according to the manufacturer’s instructions. The amount of each mRNA was quantified using a TaqMan assay with TaqMan Gene Expression Master Mix (Applied Biosystems) and gene-specific probes, primers, and standard oligos purchased from Sigma Genosys (sequences are listed in S1 Table). The quantity of mRNA was calculated by absolute standard plot of serial diluted standard oligos.

### Western blotting

siRNA-transfected HeLa cells were lysed with Cell Lysis Buffer (Cell Signaling). The lysates were separated by electrophoresis in a 7.5–15% polyacrylamide gel (DRC) and blotted onto a PVDF membrane (Millipore). The membrane was blocked with 3% BSA in TBS, incubated with primary antibody diluted in Can Get Signal Solution I (TOYOBO) overnight, incubated with HRP-linked anti-mouse or anti-rabbit IgG (Cell Signaling) diluted in TBS-T for 1 hour, and developed with ECL (GE Healthcare). Images were visualized using an ImageQuant LAS-4000 imaging system (Fujifilm).

### RIP assay

RIP assays were performed using the Magna RIP RNA-Binding Protein Immunoprecipitation Kit (Millipore) according to the manufacturer’s protocol. Normal mouse IgG (Millipore) was used as a negative control. Immunoprecipitated RNAs were reverse transcribed using SuperScript III First-Strand Synthesis System for RT-PCR (Invitrogen) according to the manufacturer’s instructions. The amount of each mRNA was quantified using the TaqMan assay.

### Immunofluorescence

Cells were washed with D-PBS and fixed with 4% paraformaldehyde in D-PBS for 15 minutes at room temperature. Fixed cells were washed with D-PBS and permeabilized with 0.1% Triton X-100 in D-PBS for 5 minutes, blocked with 5% FBS in D-PBS for 1 hour, and incubated with primary antibodies overnight. The next day, cells were washed three times with D-PBS and incubated with Alexa Fluor 488- or 594-conjugated anti-rabbit or anti-mouse IgG (Molecular Probes) for 1 hour at room temperature in the dark. After three washes, the cells were incubated with 0.5 μg/ml Hoechst 33342 (Molecular Probes) for 5 minutes and then visualized using a BZ-X700 fluorescence microscope (Keyence).

### HITS-CLIP

The standard BrdU-CLIP libraries were prepared according to the published protocol [11]. Details of the optimized CLIP protocol are described in the S1 Protocol. cDNAs were radiolabeled by using [α-^32^P]-dCTP during reverse transcription.

### Cellular fractionation

Cytoplasmic fractions of P/I-stimulated HeLa cells were prepared as previously described with slight modifications [27]. Briefly, crosslinked HeLa cells were resuspended in four times packed cell volume (PCV) of hypotonic buffer A (10 mM Tris-HCl (pH 7.5), 1 mM MgCl2, 1 mM DTT, Protease Inhibitor Cocktail (Thermo Fisher Scientific, USA; cat. #78410), and incubated on ice for 10 minutes. Samples were homogenized using a dounce homogenizer and rinsed with the same volume of buffer A, and then transferred into a 1.5 ml tube. After centrifuging at 8500 x *g* for 5 minutes at 4 °C, supernatants were harvested and then NaCl was added to a final concentration of 150 mM, and NP-40 was added to a final concentration of 0.1%. Samples were stored at -80 °C until needed.

### CLIP sequencing and bioinformatics

BrdU-CLIP and optimized CLIP libraries for HNRNPU and HNRNPC were sequenced in single-end 50 nt mode with a MiSeq system (Illumina). Sequence tags were processed using the CIMS tool [7] and aligned to the human genome (hg19) using NovoAlign (Novocraft). Crosslink-induced truncation sites (CITS) were extracted from -9 to +9 nt around the end of peak tags. CLIP-tag peak regions were annotated, and motif analysis for CITS was performed using the HOMER tool (http://homer.ucsd.edu/homer/index.html).

### mRNA sequencing and gene set overlapping

mRNA libraries were generated using the TruSeq Stranded mRNA Library Prep Kit for NeoPrep (Illumina) and sequenced in pair-end 75 nt mode with a MiSeq system (Illumina). Sequence tags were analyzed using ArrayStudio (OmicSoft). DEGs were extracted with significance at p-values < 0.05 (t-test). The significance of gene set overlapping was assessed with Fisher’s exact test.

## Results

### Optimization of HITS-CLIP method

**Fig 1:**
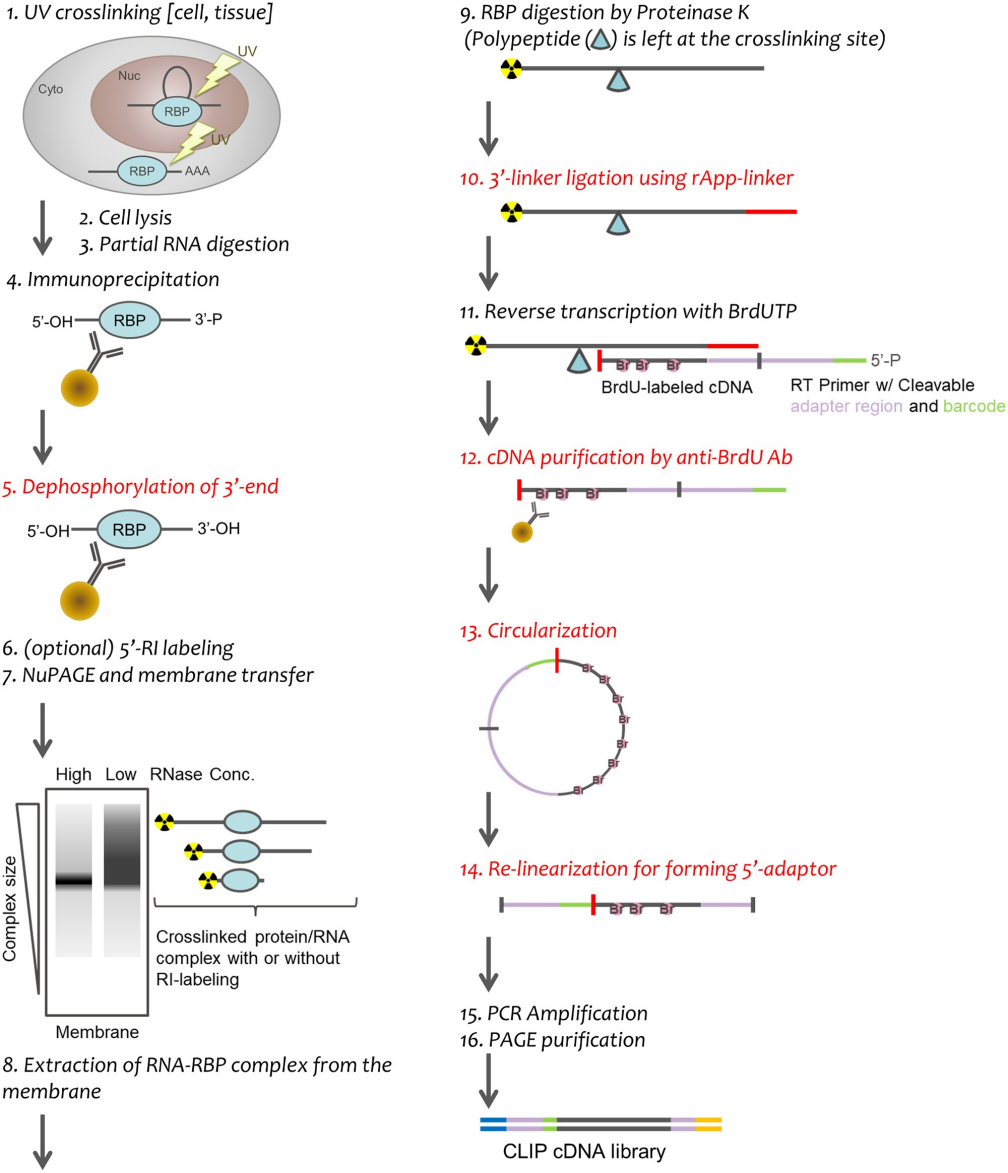
Schematic workflow of optimized CLIP. Cells are irradiated with 254 nm UV light on ice to form crosslinked RNA-RBP complexes, followed by partial RNase digestion and immunoprecipitation with RBP-specific antibodies. RNA crosslinked with RBPs is dephosphorylated for linker ligation, phosphorylated with [γ-^32^P]- ATP if necessary, separated by NuPAGE, and then transferred to a membrane. Isolated RNA is ligated with a 5'-App linker at the 3'-end and is subjected to BrdU-labeled cDNA synthesis by reverse transcription using a primer with a barcode and cleavage site. The cDNA is purified with the anti-BrdU antibody-coupled magnetic beads, circularized, digested with APE I, and amplified by PCR. Lastly, the CLIP cDNA library is subjected to size purification by PAGE. Steps modified from the original BrdU-CLIP protocol are indicated in red.

**Fig 2:**
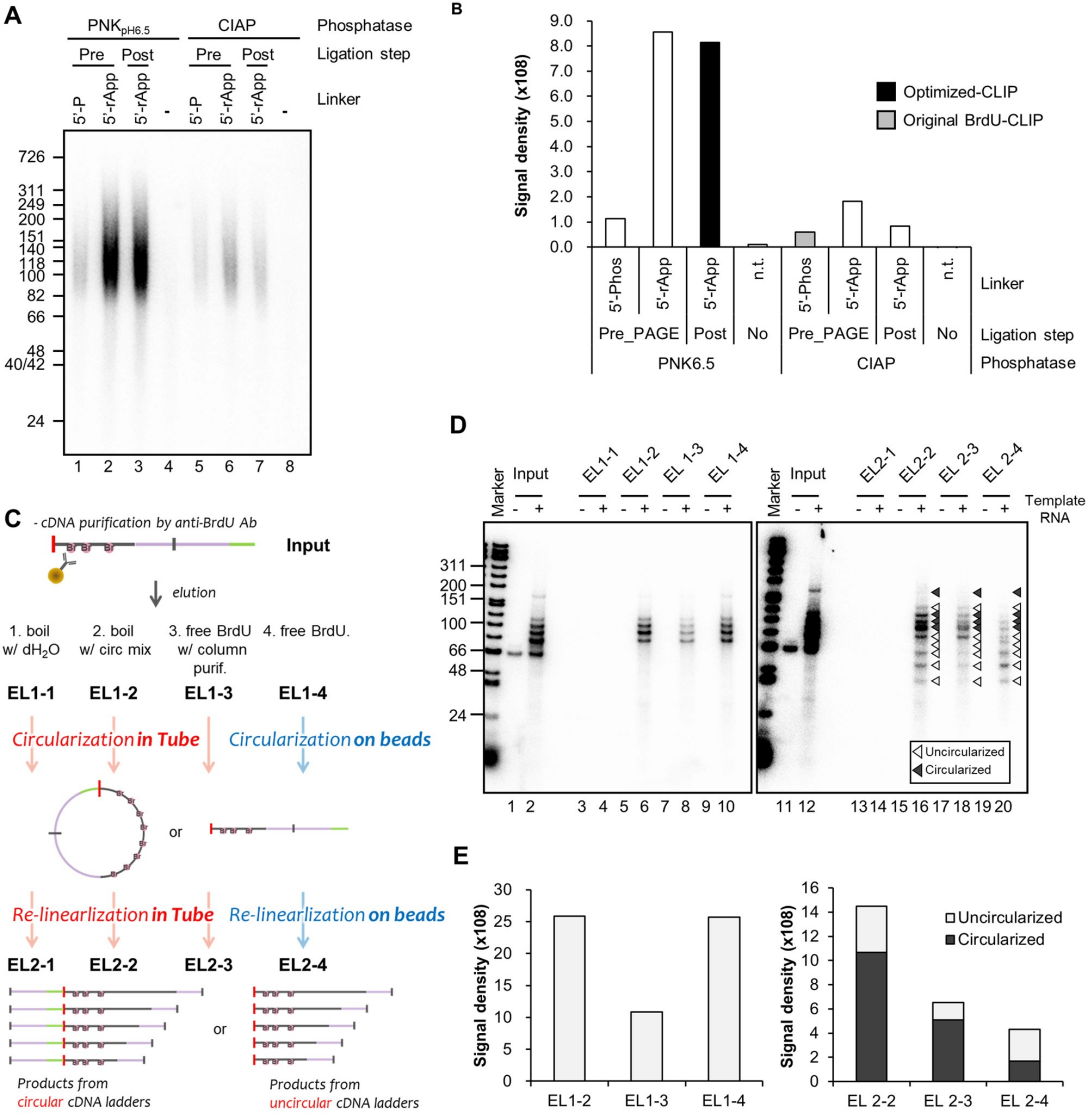
Optimization of dephosphorylation, linker ligation and circularization. (A–B) Quantification of reverse transcribed cDNA to evaluate the yield of linker-ligated RNA. Template RNA was isolated from HNRNPU-RNA, dephosphorylated with PNK or CIAP, and subsequently ligated using linkers with phosphorylated or adenylated 5’-ends. Linkers were phosphorylated or adenylated prior to polyacrylmide gel electrophoresis (PAGE) or after isolation from the membrane. cDNA that was reverse transcribed in the presence of [α-^32^P]-dCTP was purified with anti-BrdU beads and separated in a 10% TBE-urea gel (A). Signal density was quantified from the autoradiograph (B). (C) Schematics for optimization of elution during anti-BrdU purification and during circularization using RNA ladders. (D–E) Reverse transcribed cDNAs from linker-ligated RNA ladders were eluted from anti-BrdU beads as indicated in (C), and were subsequently circularized and re-linearized in tubes (EL2-1, -2, -3) or on beads (EL2-4).

We sought to optimize a more efficient CLIP method based on the BrdU-CLIP protocol [11] (Fig 1). To obtain a sufficient amount of cDNAs reverse transcribed from co-immunoprecipitated RNA fragments, we compared dephosphorylation and subsequent 3’-linker ligation reaction conditions by performing CLIP of HNRNPU in HeLa cells. Consistent with previous data [9], we obtained a 1.9-fold higher yield of radiolabeled cDNA fragments reverse transcribed from RNAs dephosphorylated by T4-PNK in pH 6.5 buffer [28] compared with RNAs dephosphorylated by alkaline phosphatase (CIAP) using a conventional 5’-phosphorylated linker (Fig 2A and 2B). Using a 5’-adenylated linker led to drastically increased yields, with a 4.7-fold increase when the linker ligation reaction was performed prior to polyacrylamide gel electrophoresis (PAGE) and a 9.8-fold increase when performed after PAGE (Fig 2A and 2B). Importantly, no differences in cDNA yield were observed between linker ligation performed before PAGE and after PAGE when the dephosphorylation reaction was performed using T4-PNK (Fig 2A, lanes 2–3 and Fig 2B), indicating that PAGE did not affect the efficiency of the linker ligation reaction. This alteration of the ligation reaction protocol shortens the time required. We confirmed that dephosphorylation by PNK with pH 6.5 buffer and subsequent ligation with a 5’-adenylated 3'-linker after PAGE isolation improved efficiency and led to a 13.6-fold increase in yield (Fig 2A, lanes 3 and Fig 2B). Next, we optimized cDNA purification and modification steps using five RNA ladders of different lengths, each ligated with a 3’-linker, as reference RNA templates (Fig 2C). We compared the elution efficiency of competitive elution with free BrdU and elution by boiling. Boiling in circularization reaction buffer recovered a similar quantity of cDNA as the original method (Fig 2D, lanes 6–10), whereas boiling in water recovered none (Fig 2D, lane 4). Competitively eluted cDNAs by free BrdU were reduced during concentration with the ssDNA/RNA Clean & Concentrator kit (Fig 2D, lane 8). We performed the circularization and re-linearization reactions on beads according to the original protocol and also in a tube as an alternative method. On-bead circularization and re-linearization reactions produced unexpected short fragments (Fig 2D, lane 20), the sizes of which correlated with digested un-circularized cDNA fragments. The ratio of fragments corresponding with circularized and un-circularized cDNAs was approximately 2 to 3, indicating that the circularization reaction was only 39% successful (Fig 2E, EL 2–4). The in-tube procedure increased the quantity of circularized cDNA 6.3-fold and improved the rate of circularization to 74% (Fig 2D, lane 16 vs 20 and Fig 2E EL2-2 (filled) vs EL2-4 (filled)). In summary, we found that using our alternative protocols of dephosphorylation with PNK, linker ligation with 5’-adenylated linker, and in-tube circularization could theoretically be expected to improve the yield 85.7-fold compared with the original BrdU-CLIP method.

### Optimized CLIP outperforms conventional BrdU-CLIP

**Fig 3:**
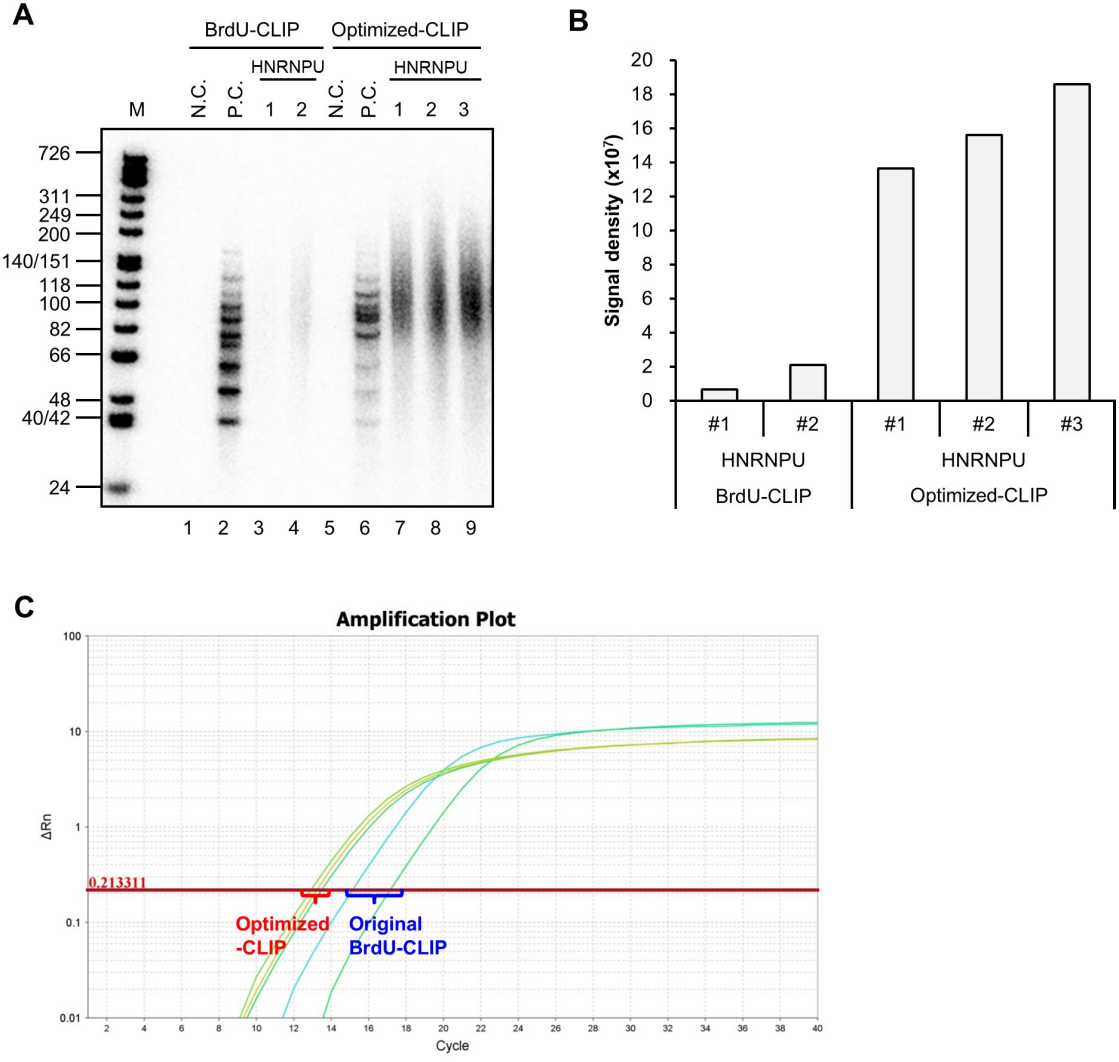
Comparison of BrdU-CLIP and optimized CLIP. Before PCR amplification, radiolabeled CLIP cDNA libraries were separated by 10% TBE-urea PAGE (A), and signal intensities were quantified (B). Signal intensities were measured between 66 and 200 nt; Averaged HNRNPU CLIP library signal intensities for optimized CLIP and BrdU-CLIP were 1.59 x 10^8^ and 1.38 x 10^7^, respectively. (C) Plot of CLIP library amplification PCR. Average Ct values for optimized CLIP and BrdU-CLIP were 13.2 and 16.2, respectively.

We evaluated how our protocol improved upon BrdU-CLIP by performing CLIP of HNRNPU. We found that prior to the amplification step, our CLIP generated an 11.5-fold increased quantity of cDNA library fragments compared with BrdU-CLIP, and with lower variance (Fig 3A and 3B). Quantitative PCR demonstrated that the cDNAs were more plentiful and less variable (Fig 3C). Moreover, replicated read rates were drastically lower in our CLIP-Seq data, possibly due to reducing the number of PCR cycles (Fig 3C; Table 1). These results show that our CLIP protocol is dramatically better at producing unique reads than BrdU-CLIP. To summarize, our optimized CLIP workflow is illustrated (Fig 1).

**Table 1:** CLIP-tags with trimming and mapping for HNRNPU BrdU-CLIP and optimized-CLIP.

Sample	Raw read	Q. Passed read		Collapse duplicate		Mapped read		Mapped uniq read	
**BrdU-CLIP rep.1**	**2,596,869**	**2,596,863**	**100.00%**	**906,248**	**34.90%**	**308,060**	**34.00%**	**281,524**	**91.40%**
**BrdU-CLIP rep.2**	**2,244,533**	**2,244,524**	**100.00%**	**1,520,956**	**67.80%**	**599,208**	**39.40%**	**581,238**	**97.00%**
**Opt-CLIP rep.1**	**1,709,708**	**1,709,700**	**100.00%**	**1,503,237**	**87.90%**	**395,673**	**26.30%**	**393,317**	**99.40%**
**Opt-CLIP rep.2**	**1,392,911**	**1,392,904**	**100.00%**	**1,273,179**	**91.40%**	**356,183**	**28.00%**	**354,453**	**99.50%**
**Opt-CLIP rep.3**	**1,587,843**	**1,587,838**	**100.00%**	**1,397,739**	**88.00%**	**367,243**	**26.30%**	**364,633**	**99.30%**

*Percentages indicate the ratio against previous process.

### Optimized CLIP is reproducible

**Fig 4:**
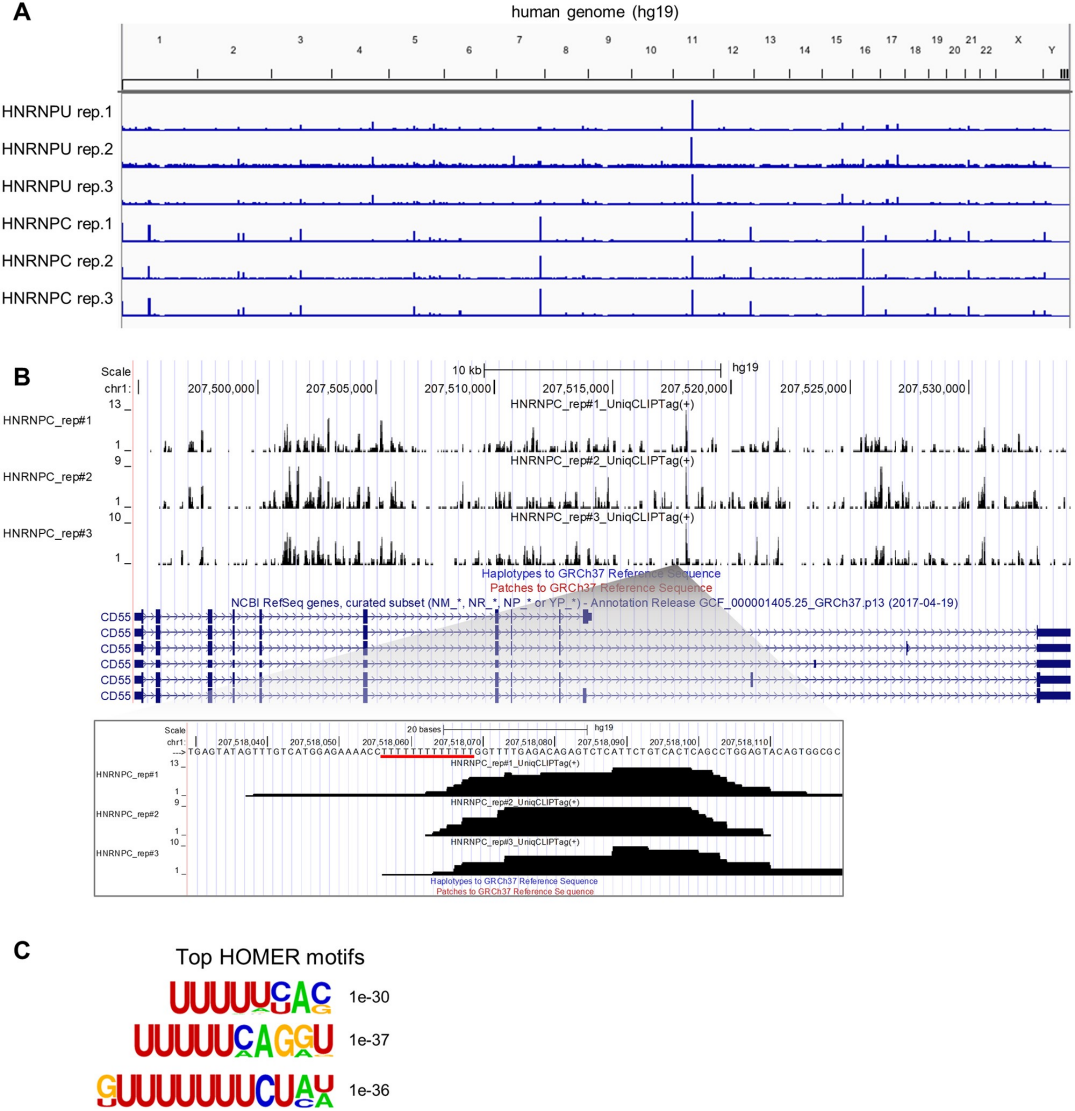
Optimized CLIP feasibility study. (A) Optimized CLIP-tag distribution across the human genome (hg19). HNRNPU and HNRNPC Optimized CLIP were performed using whole lysates from HeLa cells. CLIP-tags were visualized using the Integrative Genomics Viewer (IGV). (B) HNRNPC tag distribution at the *CD55* locus visualized using the UCSC Genome Browser, which was validated as a HNRNPC target by iCLIP and irCLIP. The highest peak at the *CD55* locus is enlarged, and the poly-U tract is underscored in red. (C) Motif analysis for CITS sites of HNRNPC CLIP. CITS sites were extracted around -9 and +9 nt of peak ends and analyzed by HOMER; motif lengths are 8, 10, and 12 nt.

HNRNPC has been well characterized by CLIP, and U-tract sequences have been identified as HNRNPC binding motifs [13]. We evaluated the performance of our CLIP method by performing in HeLa cells. We observed that the distribution of CLIP-tags over the genome was quite similar between triplicate experiments with HNRNPC, and was largely distinct from the pattern of CLIP-tags obtained using HNRNPU (Fig 4A). PCR duplicate rates of CLIP-tags were also quite low (Table 2). HNRNPC CLIP demonstrated that HRNPC binds to *CD55* RNA, a well-known HNRNPC target, and the highest CLIP peak in CD55 was adjacent to U-tract sequences (Fig 4B). Motif analysis using HOMER identified U-tract sequences as the top-scoring motifs with high significance (E-value = 1e^-30~37^) (Fig 4C). Finally, our HNRNPC CLIP successfully recapitulated previous results, confirming that our CLIP is a valid method.

**Table 2:** CLIP-tags with trimming and mapping for HNRNPC optimized-CLIP.

Sample	Raw read	Q. Passed read		Collapse duplicate		Mapped read		Mapped uniq read	
**HNRNPC rep.1**	**1,251,256**	**1251250**	**100.00%**	**1195888**	**95.60%**	**330,100**	**27.60%**	**329,741**	**99.90%**
**HNRNPC rep.2**	**1,271,549**	**1271548**	**100.00%**	**1199193**	**94.30%**	**391,704**	**32.70%**	**390,956**	**99.80%**
**HNRNPC rep.3**	**1,305,395**	**1305392**	**100.00%**	**1239696**	**95.00%**	**354,010**	**28.60%**	**353,400**	**99.80%**

*Percentages indicate the ratio against previous process.

### HNRNPU regulates IL-6

**Fig 5:**
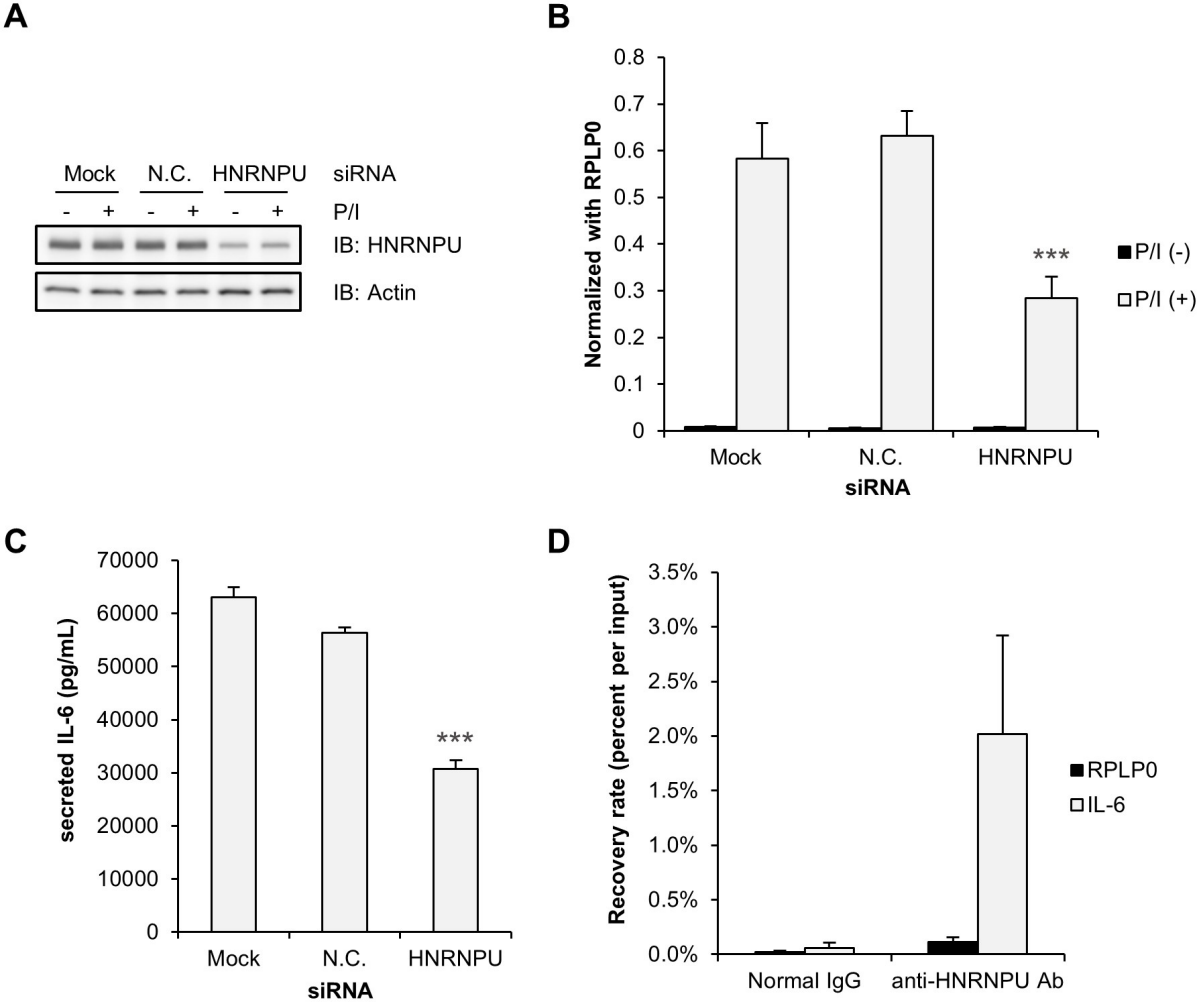
HNRNPU regulates P/I-induced *IL-6* mRNA expression. (A–C) HeLa cells were transfected with siRNAs, incubated for 72 hours, and then treated with P/I for 4 hours (B) or 24 hours (A, C) and harvested. HNRNPU protein expression was determined by Western blotting with actin as a loading control (A). *IL-6* mRNA expression was examined using a TaqMan assay and normalized with *RPLP0* (B). (C) IL-6 protein secreted in the condition medium was quantified by ELISA (R&D Systems). Statistically significant differences are marked with *** (*p* < 0.005, Welch’s t-test). (D) HeLa cell lysates that had been stimulated with P/I for 4 hours were subjected to a RIP assay using the anti-HNRNPU antibody or normal mouse IgG as a negative control. Co-immunoprecipitated mRNAs were reverse transcribed with Superscript III, and co-immunoprecipitated *IL-6* and *RPLP0* mRNA were quantified using a TaqMan assay.

Regnase-1 and Arid5a were well-known as major RNA-binding regulators of mRNAs encoding several types of cytokines during inflammation process [29–31]. In addition to these, HNRNPU has been shown to regulate *TNFα* expression in 293T cells as well as *IL-6* and *IL-1β* expression in RAW264.7 macrophage stimulated with LPS by binding the 3’-UTR, thereby stabilizing the mRNA [24, 25]. To explore cytokines targeted by HNRNPU in HeLa cells, we performed siRNA-mediated knockdown (Fig 5A) and measured the mRNA expression of several cytokines using a TaqMan assay. We found that *IL-6* mRNA was significantly decreased in HNRNPU-knockdown HeLa cells stimulated with Phorbol 12-Myristate 13-Acetate (PMA) and Ca2+ ionophore (P/I) for 4 hours (Fig 5B), and the amount of IL-6 protein secreted in the medium 24 hours after P/I induction was also decreased (Fig 5C). Using a RIP assay, we confirmed that *IL-6* mRNA was co-immunoprecipitated with HNRNPU but not with normal mouse IgG. The amount of co-immunoprecipitated *RPLP0*, a housekeeping gene, was quite small compared with *IL-6* (Fig 5D), suggesting that HNRNPU might specifically bind *IL-6* mRNA to control mRNA level and protein levels.

### HNRNPU directly binds IL-6 mRNA

**Fig 6:**
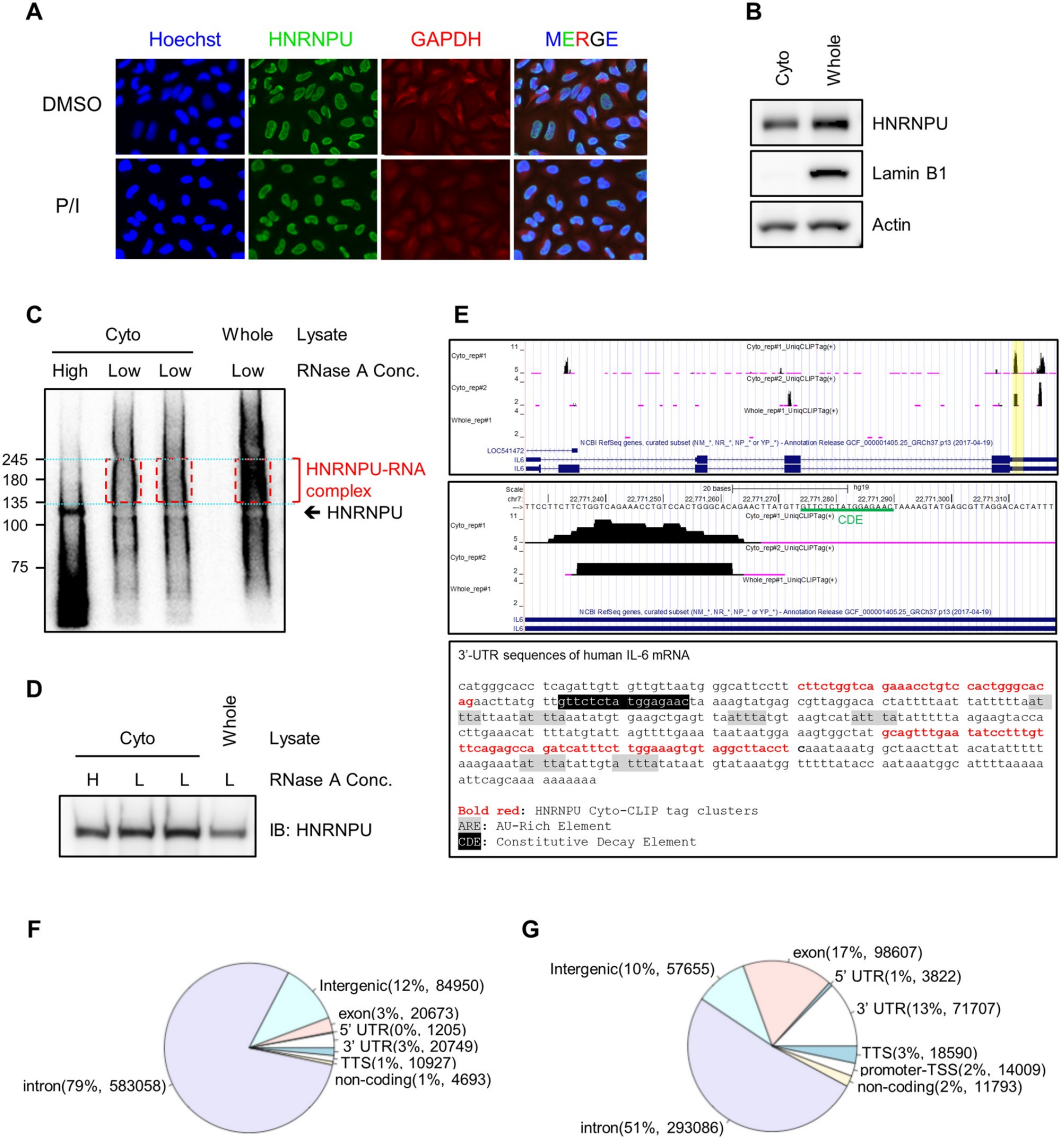
Cyto-CLIP for HNRNPU identifies a direct interaction between HNRNPU and the 3'-UTR of *IL-6* mRNA. (A) The subcellular location of HNRNPU in cells treated with P/I or DMSO for 4 hours was determined by immunofluorescence using the anti-HNRNPU antibody. The nucleus and cytoplasm were visualized with Hoechst and anti-GAPDH staining, respectively. (B) Western blotting of whole lysate (whole) and the cytoplasmic fraction (cyto) with Lamin B1 as a nuclear marker and Actin as a cytoplasmic marker. (C–D) Autoradiogram of the nitrocellulose membrane of labeled ^32^P-labeled RNA crosslinked to immunoprecipitation purified HNRNPU from cyto or whole crosslinked lysate treated with low (1:100000) or high (1:100) RNase A using an anti-HNRNPU-specific antibody (C) and detection of isolated HNRNPU by Western blotting on same membrane (D). (E) HNRNPU binds to the 3’UTR of *IL-6* mRNA in cytoplasmic fraction. The distribution of HNRNPU whole-CLIP and cyto-CLIP unique tags at the *IL-6* locus was visualized with the UCSC Genome Browser. CLIP-tag peaks are shown for whole-CLIP, cyto-CLIP replicate #1, and cyto-CLIP replicate #2. Magnified view of *IL-6* 3’UTR locus, highlighted in yellow (Top panel), including the HNRNPU binding and constitutive decay element (CDE) underscored in green (Middle panel). 3’-untranslated region (3’-UTR) sequences of human *IL-6* mRNA are shown with marks for HNRNPU cyto-CLIP, AU-rich element (ARE) and CDE. (Bottom panel) (F–G) The genomic distribution of HNRNPU whole-CLIP (F) and cyto-CLIP (G) unique tag cluster peaks are shown.

Immunocytochemistry showed HNRNPU expression exclusively in the nucleus, even after P/I treatment (Fig 6A). To check whether HNRNPU was expressed in the cytoplasm, we biochemically fractionated HeLa cell lysate and detected proteins by Western blotting. We found that HNRNPU co-fractionated with GAPDH, a cytoplasmic marker (Fig 6B). Importantly, the nuclear marker protein Lamin B1 was almost absent in cytoplasmic fraction (Fig 6B), while Histone H3 was a little leaked from nuclear fraction (S1 Fig), indicating that this biochemical fractionation was largely enriched (Fig 6B). Next, we attempted to verify direct binding of HNRNPU to *IL-6* mRNA using our CLIP with whole cell lysate (whole-CLIP) (Fig 6C and 6D), but were unable to detect any CLIP-tags on *IL-6* mRNA (Fig 6E). Importantly, only 3% of unique CLIP-tags from whole-CLIP were located in 3’-UTRs. The majority were distributed in intron sequences (79%) (Fig 6F; S2 List), suggesting a major role of HNRNPU in nuclear function. Given that interactions between RBPs and 3’-UTRs are often assumed to occur in the cytoplasm [32], we hypothesized that CLIP-tags derived from cytoplasmic fraction might be masked by those from nuclear interactions because over 90% of HNRNPU is expressed in the nucleus (Fig 6A). To investigate this possibility, we performed HNRNPU CLIP using the cytoplasmic fraction and whole cell lysate of HeLa cells, termed cyto-CLIP and whole-CLIP, respectively. We were able to detect HNRNPU-RNA interactions using cyto-CLIP (Fig 6E) and observed that the isotope-labeled signal was lower than it was with whole-CLIP, although we obtained a larger amount of immunoprecipitated HNRNPU from the cytoplasmic fraction than from the nuclear fraction (Fig 6C and 6D), suggesting that HNRNPU binds RNA more strongly in the nucleus than in the cytoplasm. Cyto-CLIP led to a markedly different distribution of CLIP-tag clusters than whole-CLIP; with cyto-CLIP, 30% of CLIP-tags were located in 3’-UTRs and coding exon, whereas with whole-CLIP, only 3–6% were in exon and 3’UTR, although the distribution of cyto-CLIP for HNRNPU was mainly located in intron sequences (51%), might be due to the leak of biochemical fractionation (Fig 6F and 6G; S1 List). Finally, we found that HNRNPU CLIP-tags were reproducibly detected in the 3’-UTR of *IL-6* mRNA (Fig 6E).

### Comprehensive identification of HNRNPU direct targets using cytosolic CLIP

**Fig 7:**
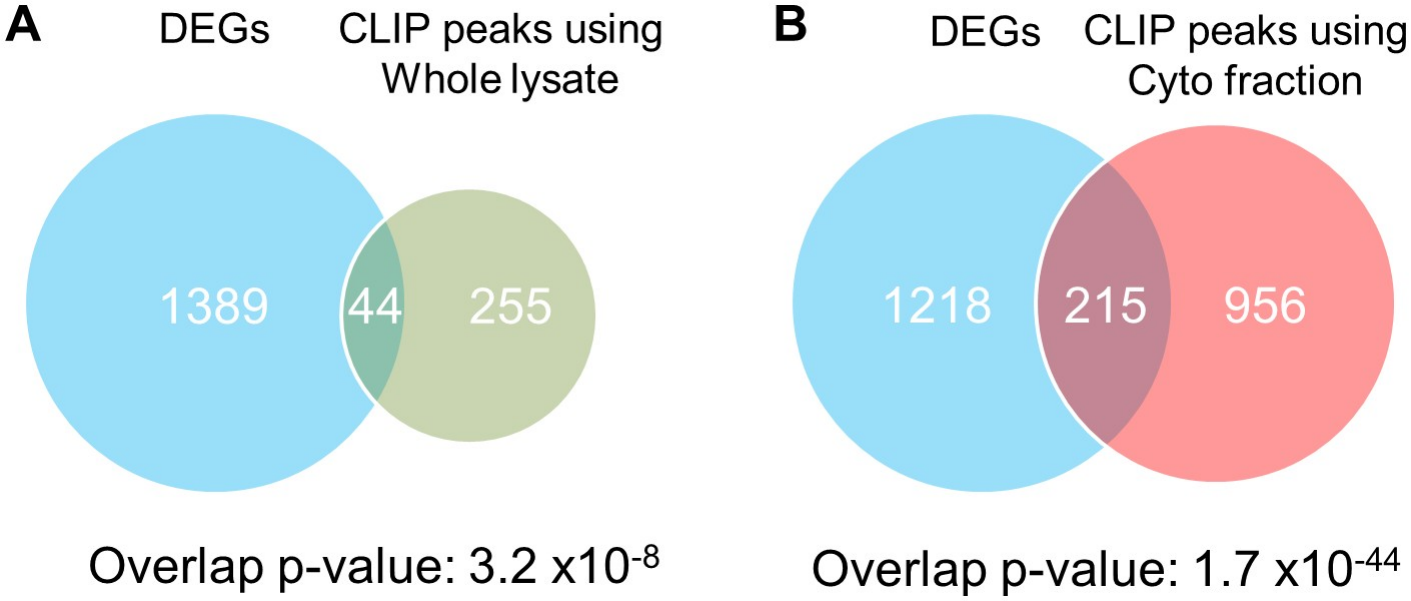
Cyto-CLIP identifies more genes directly regulated by HNRNPU than whole-CLIP. (A–B) Venn diagrams show the overlap between differentially expressed genes in HNRNPU-knockdown HeLa cells by mRNA-Seq (p-values < 0.05) and HNRNPU target genes identified by whole-CLIP (A) and cyto-CLIP (B). The overlap p-values were calculated using Fisher's exact test.

We next evaluated whether HNRNPU cyto-CLIP predicted target mRNAs. To search the direct target mRNAs of HNRNPU, we performed mRNA-seq analysis in HNRNPU-knockdown HeLa cells that had been treated with P/I. Analysis of the RNA sequencing data identified 1420 differentially expressed genes (DEGs) as HNRNPU target genes (S3 List), only 44 of which were also identified by whole-CLIP (overlap p-value = 3.2 x 10^−8^) (Fig 7A). By contrast, significantly more DEGs (214) were identified by cyto-CLIP (overlap p-value = 1.7 x 10^−44^) (Fig 7B).

## Discussion

**Fig 8:**
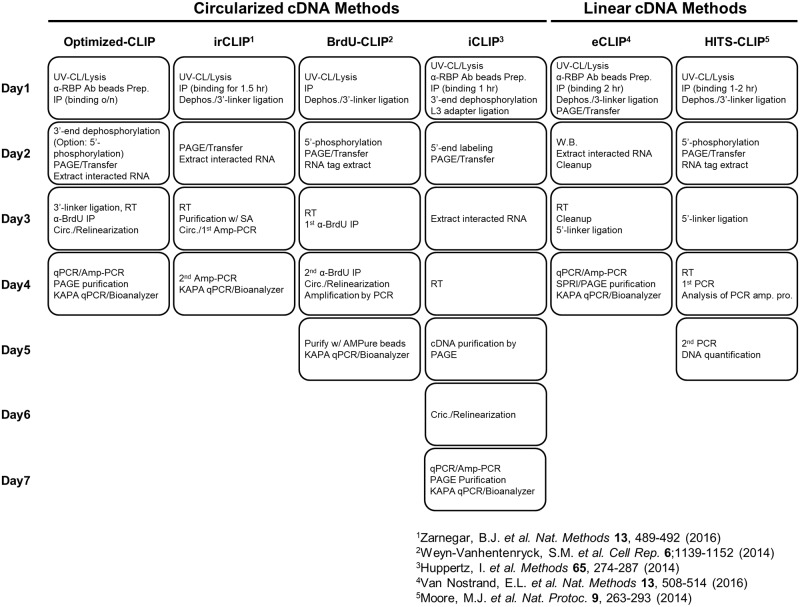
Comparison among various CLIP-seq methods. An overview of procedures and duration for currently available CLIP technologies is shown. Duration was estimated by referring to the original publications.

CLIP is the gold standard method to detect RBP-RNA interactions in living tissue and cultured cells. Because CLIP experiments are complicated to perform, several new CLIP methods have been developed to overcome technical issues and reduce time-consuming steps, as well as to identify precise protein-RNA interaction sites at single-nucleotide resolution (Fig 8). We improved CLIP method with the goal of creating a rapid, highly reproducible assay that involves minimal handling. Additionally, our CLIP experiment includes a universal positive control to measure the efficiency of enzyme and nucleotide purification steps. We validated our CLIP method by performing an experiment with HNRNPC in HeLa cells and obtained highly reproducible results. Finally, we used our CLIP strategy to capture a previously uncharacterized interaction between HNRNPU and *IL-6* mRNA in the cytoplasm that controls mRNA stability.

To generate high-yield CLIP libraries, we used isolated RNA fragments as a universal positive control for determining how efficiently the 3’-linker attaches to an RNA-RBP complex. In our optimized method, linker-ligated RNA is efficiently produced by performing a low-pH PNK-dependent dephosphorylation reaction concurrently with ligation using a 5’-adenylated 3’-linker, which also reduces the incubation time (Fig 2A and 2B). We found that the circularization on beads was inefficient and led to loss of cDNA (Fig 2D and 2E). In particular, the decreased amount of cDNA after circularization and re-linearization could be due to partial dissociation from beads caused by the relatively high temperature (65 °C) used for the circularization reaction. Indeed, we observed radio counts derived from isotope-labeled cDNA in the supernatant after a circularization reaction. We therefore opted to circularize and re-linearize cDNA in tubes rather than on beads. Supporting our observation, PAPERCLIP, another recently reported CLIP method, also does not use beads [33]. A universal positive control is useful for optimizing the efficiency of CLIP library generation and for identifying experimental errors step-by-step, especially for those who are unfamiliar with CLIP methods (Fig 2C). We further performed an additional CLIP experiment using HNRNPC protein, a nuclear RBP that does not shuttle between the nucleus and cytoplasm, to confirm the precise identification of binding sites and determine reproducibility (Fig 4 and Table 2) [34].

CLIP has been performed with radioisotope (RI) labeling to detect RNA-RBP complexes. Although we used RI labeling in our method, other methods such as eCLIP and irCLIP do not employ RI labeling [12, 13]. Once we confirmed the position of the RNA-RBP complex relative to pre-stained molecular weight markers in a polyacrylamide gel, we successfully performed CLIP without RI labeling. We recommend checking the position of RI-labeled RNA-RBP complexes on a polyacrylamide gel when first performing CLIP experiments to optimize IP conditions such as RNase concentration, lysate volume, and antibodies.

We conducted optimized CLIP experiments with HNRNPU, a multi-functional protein that contains DNA- and RNA-binding motifs and, unlike HNRNPC, shuttles between the nucleus and cytoplasm. We observed that HNRNPU is mostly localized in the nucleus, but a subcellular fractionation assay confirmed cytoplasmic localization as well (Fig 6). Considering the multiple cellular localizations and functions of HNRNPU, it would be difficult to map out protein-RNA interactions in each specific cellular context, similar to other nucleocytoplasmic shuttling RBPs. Such situations would benefit from an optimized CLIP method that utilizes a smaller amount of RNA, such as would be obtained from a subcellular fraction, specific organelle, or particular subcellular region such as the dendrite or axon of a neuron. In the present study, we validated the interaction between HNRNPU and the 3’-UTR of *IL-6* mRNA using cyto-CLIP, a variant of CLIP method, using only the cytoplasmic fraction, despite being unable to observe the interaction with our CLIP experiment using whole cell lysate (Fig 6E). The CLIP-tag distribution showed that over 90% of CLIP-tags located in introns and intergenic regions (Fig 6F), reflecting the nuclear localization of HNRNPU even when cells were stimulated with P/I (Fig 6A). Interactions between the 3’-UTRs of mRNAs and RBPs are thought to occur in the cytoplasm but not in the nucleus, for example, the main CLIPed distribution of cytoplasmic neuronal ELAV proteins is 3’UTR [35]. Similar to our observations, CLIP-Seq using subcellular fractions also identified different CLIP-tags for the nucleocytoplasmic shuttling RBP SF2/ASF [36]. Our CLIP strategy is useful for validating specific protein-RNA interactions even if they constitute a minor fraction of a distributed RBP, such as the interaction between HNRNPU with the 3’-UTR of *IL-6*. Next, we could approach more comprehensive understanding of mRNA targeting and molecular mechanisms for HNRNPU using a CLIP technology combined with another layer of RNA mapping such as transcriptome. First, by using cyto-CLIP in conjunction with mRNA-Seq in HNRNPU-knockdown cells, we found 213 DEGs that were direct HNRNPU target genes, a significantly greater number than were found using CLIP method and mRNA-Seq (Fig 7). Second, we still poorly understood an HNRNPU dependent molecular mechanism in mRNA stability control. Lots of RBPs are reported to be involved in *IL-6* mRNA stability control, mediating constitutive decay elements (CDE) and AU-rich elements (ARE) in this 3’UTR [37]. Interestingly, our HNRNPU CLIP sites in *IL-6* mRNA were located in close to CDE region (Fig 6), which Regnase-1 (ZC3H12A), Roquin1/2 (RC3H1/2) and Arid5a might recognize [30, 31, 37]. Their functional involvement of HNRNPU with these regulators would warrant the further study.

In conclusion, our data show that optimized CLIP is an efficient method suitable for use in conjunction with cellular fractionation and may provide a general means of analyzing complex RBP functions in specific localized RNA, RNA containing organelles and tissues.

## Supporting information

S1 List Cyto-CLIP_annotation. (TXT)

S2 List Whole-CLIP_annotation. (TXT)

S3 List DEGs_in_HNRNPU-KD. (TXT)

S1 Table Primer_Oligo. (PDF)

S1 Protocol Optimized-CLIP_Protocol. (PDF)

S1 Fig Fractionation check by Histone H3. Western blotting of whole lysate (whole) and the cytoplasmic fraction (cyto) with Histone H3 (Related to Fig 6B).
(TIF)

S2 Fig Original uncropped gel images. (TIF)
