# Supplementary material for: Analysis of the nucleocytoplasmic shuttling RNA-binding protein HNRNPU using optimized HITS-CLIP method
Source: PLoS One. 2020 Apr 17;15(4):e0231450. doi: 10.1371/journal.pone.0231450 (PMC7164624; doi:10.1371/journal.pone.0231450)
Supplement: S1 Table — (PDF) [file pone.0231450.s004.pdf]

Supplementary Table: Primer and Oligo sequences for TaqMan assay

Gene	Name	Sequence
IL-6	IL-6 TaqMan probe	AGATGCAATAACCACCCCTGACCCAACC [5'-FAM, 3'-TAMRA]
	IL-6 fwd primer	GTACAAAAGTCCTGATCCAGTTCC
	IL-6 rev primer	TCGTCAGCAGGCTGGCATT
	IL-6 std oligo	GATGAGTACAAAAGTCCTGATCCAGTTCCTGCAGAAAAAGGCAAAGAA<br>TCTAGATGCAATAACCACCCCTGACCCAACCACAAATGCCAGCCTGCT
RPLP0	RPLP0 TaqMan probe	TTGTCTGCTCCCACAATGAAACATTTCGGA [5'-HEX, 3'-TAMRA]
	RPLP0 fwd primer	CAGGGCGACCTGGAAGTCC
	RPLP0 rev primer	GCTGCATCTGCTTGGAGCC
	RPLP0 std oligo	GAAGACAGGGCGACCTGGAAGTCCAACTACTTCCTTAAGATCATCCAA<br>CTATTGGATGATTATCCGAAATGTTTCATTGTGGGAGCAGACAATGTG<br>GGCTCCAAGCAGATGCAGCAGATC
