# Supplementary material for: Analysis of the nucleocytoplasmic shuttling RNA-binding protein HNRNPU using optimized HITS-CLIP method
Source: PLoS One. 2020 Apr 17;15(4):e0231450. doi: 10.1371/journal.pone.0231450 (PMC7164624; doi:10.1371/journal.pone.0231450)
Supplement: S1 Protocol — (PDF) [file pone.0231450.s005.pdf]

Optimized-CLIP Protocol

Optimized-CLIP Standard Protocol v1.0 (2020/02/027) by Masato Yugami (masato.yugami@takeda.com)

Comments/Logs

Steps

Day 0*:	I. UV crosslinking	**Day 0 procedure could be done on the same day for that of Day 1*
Day 1:	II. RNase treated lysate preparation	
	III. Immunoprecipitation (Binding)	
Day 2:	IV. Immunoprecipitation (Wash)	
	V. 3'-end dephosphorylation	
	VI. (5'-end labeling)	
	VII. PAGE & Membrane transfer	
	VIII. RNA-RBP complex extraction	
Day 3:	IX. 3'-linker ligation & column purification	
	X. cDNA synthesis	
	XI. cDNA purification by IP	
	XII. Circularization & Re-linearization	
	IXX. Phenol/Chloroform extraction & Ethanol precipitation	
Day 4:	XX. Amplification PCR	
	XXI. Size purification by PAGE	
Day 5:	XX. Generated CLIP library QC check (Bioanalyzer & qPCR)	
	XXI. Sequence with MiSeq	
Day x:	S-I. 5'-adenylated 3'-linker preparation	

Materials

Enzyme

RQ1 DNase (M610A, Promega)

RNase A (70194Y, Affymetrix)

T4 Polynucleotide Kinase (PNK) (M0201, NEB)

Proteinase K (AM2546, Ambion)

T4 RNA Ligase 1 (M0204, NEB)

Superscript III (18080-044, Invitrogen)

RNase H (18021-014, Invitrogen)

CircLigase II ssDNA Ligase (CL9021K, Epicentre)

APE 1 (M0282, NEB)

Reagent

Halt Protease Inhibitor Cocktail (100x) (78430, Thermo)

Dynabeads Protein G (10004D, Invitrogen)

RNaseOUT (10777-019, Invitrogen)

100 mM dNTP Set (10297-018, Invitrogen)

10 mM BrdUTP (B21550, Invitrogen)

GlycoBlue Coprecipitant (AM9515, Ambion)

Phusion High-Fidelity PCR Master Mix (M0531, NEB)

SYBR Green I (S7563, Invitrogen)

SYBR Gold (S11494, Invitrogen)

MicroSpin G-25 Column (95017-621, GE Healthcare)

Nanosep MF column (ODM45C33, PALL)

Marker

ΦX174 DNA/HinfI Dephosphorylated Markers (E3511, Promega)

Small RNA II Ladder Marker (DM192, BioDynamics Lab)

Antibody

Normal mouse IgG (12-370, Millipore)

Normal rabbit IgG (12-371, Millipore)

Anti-BrdU antibody, clone IIB5 (MAB3222, Millipore)

Solution

Acid-Phenol:Chloroform (AM9720, Ambion)

Phenol:Chloroform:IAA (AM9730, Ambion)

50x Denhardt’s solution (750018, Invitrogen)

Kit

5’ DNA Adenylation Kit (E2610, NEB)

RNeasy MinElute Cleanup Kit (74204, Qiagen)

MinElute Gel Extraction Kit (28604, Qiagen)

Buffers

Beads Prep Buffer
1x PBS

0.02% Tween-20

Whole Lysis Buffer
1x PBS

0.1% SDS

0.5% NaDOC

0.5% NP-40

High-salt Wash Buffer
5x PBS

0.1% SDS

0.5% NaDOC

0.5% NP-40

PNK Buffer
50 mM Tris-HCl (pH 7.5)

10 mM $MgCl_2$

0.5% NP-40

5x PNK Phosphase Buffer (pH 6.5)
350 mM Tris-HCl (pH 6.5)

10 mM $MgCl_2$

0.5% NP-40

PNK+EGTA Buffer
50 mM Tris-HCl (pH 7.5)

20 mM EGTA

0.5% NP-40

PK Buffer
100 mM Tris-HCl (pH 7.5)

50 mM NaCl

10 mM EDTA

2xIP Buffer
0.5x SSPE

2 mM EDTA

0.1% Tween-20

Nelson Low-salt Buffer
15 mM Tris-HCl (pH 7.5)

5 mM EDTA

Nelson Stringent Buffer
15 mM Tris-HCl (pH 7.5)

5 mM EDTA

120 mM NaCl

25 mM KCl

2.5 mM EGTA

1% Triton X-100

1% NaDOC

0.1% SDS

CircLigase Wash Buffer
33 mM Tris-Acetate (pH 8.0)

66 mM KOAc (pH 5.5)

Diffusion Buffer
500 mM Ammonium acetate

10 mM Magnesium acetate

1 mM EDTA (pH 8.0)

0.1% SDS

Oligos

We purchased all oligo nucleotides from Sigma Genosys (http://www.genosys.jp)

3’ Linker RNA

RL3: 5'-p_UCGUAUGCCGUCUUCUGCUUG_N(6)-3’

[p=Phosphorylation, N(6)=amino C6 linker]

Barcoded RT Primer DNAs

RT1_GCAT: 5’-p_GNNNNNNNNG-ATGC-GATCGTCGGACTGTAGAACTCT_α_CAAGCAGAAGACGGCATACGA-3’

RT2_GTCA: 5’-p_GNNNNNNNNG-TGAC-GATCGTCGGACTGTAGAACTCT_α_CAAGCAGAAGACGGCATACGA-3’

RT3_ACTG: 5’-p_GNNNNNNNNG-CAGT-GATCGTCGGACTGTAGAACTCT_α_CAAGCAGAAGACGGCATACGA-3’

RT4_AGCT: 5’-p_GNNNNNNNNG-AGCT-GATCGTCGGACTGTAGAACTCT_α_CAAGCAGAAGACGGCATACGA-3’

RT5_TGAC: 5’-p_GNNNNNNNNG-GTCA-GATCGTCGGACTGTAGAACTCT_α_CAAGCAGAAGACGGCATACGA-3’

RT6_TCGA: 5’-p_GNNNNNNNNG-TCGA-GATCGTCGGACTGTAGAACTCT_α_CAAGCAGAAGACGGCATACGA-3’

[N=mix DNA, p=Phosphorylation, α=dSpacer]

PCR Primer DNAs

DSFP5-PE: 5'-AATGATACGGCGACCACCGAGATCTACACCAGGTTCAGAGTTCTACAGTCCGACG-3’

SP3: 5'-CAAGCAGAAGACGGCATA-3’

Procedure

Day 0

I. UV cross-linking of tissue/cell cultures

For tissues (this case for mouse brain):

1. Harvest tissue from embryonic brain and let it sit in ice-cold HBSS
2. Add 10x volume of HBSS and triturate tissue by 3-times pipetting using a 5 mL pipet
3. Repeat to triturate by 3-times pipetting using the previously used 5 mL pipet added a 100 µL micropipette tip at the point
4. Transfer tissue suspension in a 10-cm dish
5. Irradiate UV on ice 3 times for 400 mJ/cm^2 ([total 1,200 mJ/cm^2]
6. Recover the suspension in 15 mL tubes
7. Centrifuge at 1,300x*g*, 4 ℃, 5 min
8. Remove supernatant
9. Resuspend in 1 mL of HBSS, transfer in a 1.5 mL micro tube
10. Centrifuge at 2,500x*g*, 4 ℃, 5 min
11. Discard supernatant and freeze the cell pellet at -80 ℃ until use

For cell cultures:

1. Grow cells in a 10-cm or 15-cm dish
2. Irradiate UV on ice twice for 300 mJ/cm^2 [total 600 mJ/cm^2]
3. Remove the medium, wash once with ice-cold 1x PBS
4. Scrape the cells in 1x PBS, transfer the cell suspension to a 1.5 mL micro tube
5. Centrifuge at 2,500x*g*, 4 ℃, 5 min
6. Discard supernatant and freeze the cell pellet at -80 ℃ until use

Day 1

II. Immunoprecipitation (Binding)

RNase-treated lysate preparation:

1. Add 1 mL of ice-cold Whole Lysis Buffer to a stored cell pellet
2. Add 10 µL of 100x Protease Inhibitor Cocktail, mix well
3. Sit on ice for 10 min to lyse
4. (Optional) Sonicate in Bioruptor at 'low' density, 4 ℃, 5 cycles with alternating 30 sec on / 30 sec off
5. Add 15 µL of DNase, incubate in Thermomixer at 1000 rpm at 37 ℃ for 5 min
6. Add 6.25 µL of 1:100 (High) or 1:10000 (Low)* RNase A diluted with Whole Lysis Buffer, incubate in Thermomixer at 1,000 rpm at 37 ℃ for 5 min **need to optimize because the desirable concentration is varied depending on cell/tissue spices, cell/tissue density, target RBPs, etc…*
7. Ultracentrifuge at 30,000 rpm at 4 ℃ for 20 min using TLA120.2 rotor (Beckman Coulter)
8. Collect supernatant as "RNase-treated lysate"
9. (Optional) If western blot analysis is performed, collect 10 µL (1%) of the lysate as a "Input" sample

Antibody-coupled beads preparation:

1. Suspend Dynabeads Protein G thoroughly by vortex
2. Dispense 50 µL per sample of Dynabeads Protein G in a 1.5 mL micro tube

3. Stand on a magnetic stand, remove supernatant
4. Wash the beads 3 times with Beads Prep Buffer
5. Add 5 µg of anti-RBP or control antibody in 100 µL of washed beads
6. Rotate the beads at room temp for 1 hr
7. Wash the beads 3 times with 1 mL of Beads Prep Buffer
8. Stand on a magnetic stand, remove supernatant
9. Add the whole volume of "RNase-treated lysate" to the beads, rotate at 4 ℃ for overnight

Day 2

III. Immunoprecipitation (Wash)

1. Wash twice with 1 mL of ice-cold Whole Lysis Buffer
2. Wash twice with 1 mL of ice-cold High-salt Wash Buffer
3. Wash twice with 1 mL of ice-cold PNK Buffer

IV. Dephophorylation by $PNK_{pH6.5}$ (On-Beads)

PNK-dephoshorylation mix

5x PNK Phosphatase Buffer (pH 6.5)	4 µL
PNK	1 µL
RNaseOUT	1 µL
RNase-free water	14 µL

1. Add 20 µL of PNK dephosphorylation mix to each sample, incubate in Thermomixer at 1000 rpm for 15 sec every 2 min, 37 ℃, 20 min
2. Wash once with 1 mL of ice-cold PNK Buffer
3. Wash once with 1 mL of ice-cold PNK+EGTA Buffer
4. Wash twice with 1 mL of ice-cold PNK Buffer
5. (Optional) If western blot analysis is performed, resuspend in 1 mL of PNK buffer, and collect 100 µL (10%) of the lysate as a "IP" sample.

V. 5'-end labeling (On-Beads)

We usually execute in optimization experiments (until step VI), but skip this step in CLIP-library generation.

PNK labeling mix

10x PNK reaction buffer	4 µL
PNK	2 µL
[γ-^{32}P] ATP	2 µL
RNase-free water	32 µL

6. Add 40 µL of PNK labeling mix to each sample, incubate in Thermomixer at 1000 rpm for 15 sec every 2 min, 37 ℃, 20 min
7. (Option) Add 10 µL of 1 mM ATP, prolong incubation in Thermomixer at 1000 rpm for 15 sec every 2 min, 37 ℃, 5 min
8. Wash once with 1 mL of ice-cold PNK Buffer
9. Wash once with 1 mL of ice-cold High-salt Wash Buffer
10. Wash twice with 1 mL of ice-cold PNK Buffer

VI. SDS-PAGE & nitrocellulose transfer

1. Stand on a magnetic stand, remove supernatant
2. Add 20 µL of 1x NuPAGE LDS Sample Buffer diluted with PNK Buffer, denature in Thermomixer at 1000 rpm, 70 ℃, 10 min

3. Stand on a magnetic stand, load the supernatant on 12-well NuPAGE mini gel [4-12% Bis-Tris or 3-8% Tris-Acetate mini gel]*

   **dependent on Mw of a target RBP*

4. Run at 150 V, 60 min in cold showcase [with corresponding buffer to the gel: 1x MOPS or 1x TA Buffer]
5. Transfer gel to nitrocellulose membrane in 1x NuPAGE Transfer Buffer with 10% Methanol at 30V, 1 hour in cold showcase
6. Rinse the membrane with 1x PBS, wrap and expose to BAS imaging plate in a cassette for 1-2 hours
7. Visualize autoradiogram with a bioimaging analyzer (GE Typhoon FLA 7000)

VII. RNA Isolation & Purification

1. Print out autoradiogram in actual size, and mark the position of smear RNA-RBP complex bands on a weighing paper
2. Excise the bands on the membrane over marked weighing paper with a clean scalpel
3. Cut into small pieces, put into a 1.5 mL micro tube
4. Preheat 4 mg/mL protease K diluted with PK Buffer, 37 ℃, 20 min
5. Add 200 μL of protease K solution, incubate in Thermomixer at 1000 rpm, 37 ℃, 20 min
6. Add 200 μL of 7M Urea in PK Buffer, incubate in Thermomixer at 1000 rpm, 37 ℃, 20 min
7. Add 400 μL of Acid-Phenol:Chloroform, mix by vortex mixer, incubate in Thermomixer at 1000 rpm, 37 ℃, 20 min
8. Centrifuge at 20,000x*g*, room temp., 5 min
9. Transfer aqueous phase to a new 1.5 mL micro tube.
10. Add 50 μL of 3M NaOAc (pH5.5), 1 mL of 1:1 mix of ethanol and isopropanol, 1.5 μL of GlycoBlue
11. Precipitate, -30 ℃, overnight

Day 3

3'-linker Ligation & Cleanup

3'-linker Ligation:

1. Centrifuge the RNA at 20,000x*g*, 4 ℃, 10 min
2. Wash twice with 1mL of cold 75% ethanol
3. Dry up the pellet, resuspend in 10 μL of RNase-free water*

Here, we also use 10 μL of 10,000-fold diluted Small RNA II ladder (DM192) as positive control

Ligation mix

20 μM rApp-3'-linker	1 μL
10x T4 RNA Ligase Buffer	2 μL
RNaseOUT	0.5 μL
T4 RNA Ligase 1	1 μL
RNase-free water	1.5 μL

4. Add 6 μL Ligation mix and 4 μL preheated PEG400, incubate, 37 ℃, 1 hour

Cleanup 3' linker-ligated RNA using RNeasy MinElute Cleanup Kit:

1. Add 80 μL RNase-free water in a post ligation reaction tube
2. Add 350 μL Buffer RLT, and mix well
3. Add 700 μL 100% ethanol, and mix well
4. Transfer 700 μL of the sample to an RNeasy MinElute spin column placed in a 2 mL collection tube
5. Centrifuge at 10,000x*g*, room temp., 15 sec, and then discard the flow-through
6. Transfer the remaining sample, repeat to centrifuge and discard
7. Place the spin column in a new 2 mL collection tube

8. Add 500 µL Buffer RPE
9. Centrifuge at 10,000x*g*, room temp., 15 sec, and then discard the flow-through
10. Add 500 µL 80% ethanol
11. Centrifuge at 10,000x*g*, room temp., 15 sec, and then discard the flow-through
12. Place the spin column in a new 2 mL collection tube, open its lid
13. Centrifuge at 20,000x*g*, room temp., 5 min
14. Place the spin column in a new 1.5 mL collection tube
15. Add 10 µL RNase-free water directly to the center of the spin column membrane
16. Centrifuge at 20,000x*g*, room temp., 1 min
17. Recover the flow-through (eluted 3'-linker ligated RNA)

VIII. cDNA synthesis & Immunoprecipitation

<u>Anti-BrdU antibody-coupled beads preparation:</u>

1. Suspend Dynabeads Protein G thoroughly by vortex
2. Dispense 25 µL per sample of Dynabeads Protein G in a 1.5 mL micro tube
3. Stand on a magnetic stand, remove supernatant
4. Wash the beads 3 times with Beads Prep Buffer
5. Add 125 µL of 5x Denhardt's solution diluted with Beads Prep Buffer
6. Rotate the beads at room temp. for 1 hr
7. Wash the beads 3 times with 1 mL of Beads Prep Buffer
8. Add 2.5 µg of anti-BrdU antibody, 5 µL of 50x Denhardt's solution, and Beads Prep Buffer up to 50 µL
9. Rotate the beads at room temp. for 1 hr
10. Wash the beads 3 times with 1 mL of 1x IP Buffer

<u>Reverse Transcription:</u>

1. Transfer 8 µL of 3'-Linker ligated RNA to a PCR tube (on ice)
2. Add 1 µL of 2 µM barcoded RT-primer

**<u>RT mix I</u>**	
5x RT buffer	4 µL
8.2 mM dATP	1 µL
8.2 mM dGTP	1 µL
8.2 mM dCTP	1 µL
8.2 mM BrdUTP	1 µL

3. Add 8 µL of RT mix I
4. Denature at 75 ℃ for 3 min, ramp down to 50 ℃ and hold

**<u>RT mix II</u>**	
0.1 M DTT	1 µL
RNaseOUT	1 µL
Superscript III	1 µL

5. Add 3 µL of RT mix II
6. Incubate on Thermal Cycler

[Step 1] 50 ℃	45 min
[Step 2] 55 ℃	15 min
[Step 3] 85 ℃	5 min
[Step 4] 37 ℃	hold

7. Add 1 µL of RNase H

8. Incubate at 37 ℃ for 20 min
9. Add 19 µL of DNase-free water
10. Pass through G-25 column by centrifugation at 3,000 rpm for 1 min

cDNA purification with anti-BrdU antibody-coupled beads:

1. Measure volume of cDNA, add DNase-free water up to 60 µL
2. Add 75 µL of 2x IP Buffer and 15 µL of 50x Denhardt's solution
3. Incubate at 70 ℃ for 3 min, and then put on ice for 3 min
4. Add to prepared the tube of Anti-BrdU antibody-coupled beads
5. Rotate the beads at room temp. for 1 hr
6. Wash the beads 1 time with 1 mL of 1x IP Buffer containing 5x Denhardt's solution
7. Wash the beads 2 times with 1 mL of Nelson Low-salt Buffer containing 1x Denhardt's solution
8. Wash the beads 2 times with 1 mL of Nelson Stringent Buffer containing 1x Denhardt's solution
9. Wash the beads 2 time with 1 mL of CircLigase Wash Buffer

**Circ Reaction Mix**	
10x Circ reaction buffer	2 µL
5 M Betaine	4 µL
50 mM $MnCl_2$	1 µL
DNase-free water	13 µL

10. Add 20 µL of Circ Reaction Mix
11. Denature at 98 ℃ for 3 min
12. Stand on a magnetic stand, recover the supernatant* **You can stop here and store the sample at -30 ℃*

IX. Circularization & Re-linearization

Circularization:

**Circ Enzyme Mix**	
0.1 M DTT	0.2 µL
CircLigase	0.5 µL
DNase-free water	0.3 µL

1. Add 1 µL of Circ Enzyme Mix to the sample
2. Incubate at 60 ℃ for 1 hour
3. Incubate at 80 ℃ for 10 min to inactivate the enzyme
4. Let sit on ice for cool down

Re-linearization:

**APE1 Reaction Mix**	
10x APE1 reaction buffer	10 µL
APE1	1 µL
DNase-free water	69 µL

5. Add 80 µL of APE1 Reaction Mix
6. Incubate at 37 ℃ for 1 hour

X. Phenol/Chloroform extraction & Ethanol precipitation

Phenol/Chloroform extraction:

1. Add 200 µL of Phenol:Chloroform:Isoamyl Alcohol (25:24:1, pH 7.9), mix by vortex mixer

2. Centrifuge at 20,000x*g*, room temp., 5 min
3. Transfer aqueous phase to a new 1.5 mL micro tube.
4. Add 20 µL of 3M NaOAc (pH5.5), 500 µL of 100% ethanol, 1.5 µL of GlycoBlue
5. Precipitate, -30 ℃, overnight

Day 4

XI. Amplification PCR

1. Centrifuge the RNA at 20,000x*g*, 4 ℃, 10 min
2. Wash twice with 1mL of cold 70% ethanol
3. Dry up the pellet, resuspend in 15 µL of DNase-free water
4. Transfer 12.5 µL* of cDNA library to a PCR tube **For check success of cDNA lib. prep., the rest cDNA was analyzed by TaqMan PCR in advance*

Phusion PCR Reaction Mix

2x Phusion Master Mix	12.5 µL	
20 µM DSFP5-PE primer	0.5 µL	
20 µM SP3 primer	0.5 µL	
50x SYBR Green I*	0.5 µL	**dilute 10,000x stock to 50x in dH_2O*

5. Add 12.5 µL of Phusion PCR Reaction Mix
6. Execute PCR reaction in real-time PCR machine

[Step 1] 98 ℃ 30 sec
Step 2-4: Cycle reaction (cycle # depending on the cDNA library)
[Step 2] 98 ℃ 10 sec
[Step 3] 65 ℃ 15 sec
[Step 4] 72 ℃ 20 sec
[Step 5] 25 ℃ hold

XII. PAGE Purification

TBE-PAGE:

1. Add 5µL of 6x Loading Dye to a PCR product, mix
2. Load the sample on 7.5% TBE Gel
3. Run at 200 V for 40 min in TBE buffer
4. Stain the gel with SybrGold 10000-fold diluted with TBE buffer for 5 min
5. Visualize on a blue-light transilluminator, excise the library smear bands on the gel [typically 120-200 bp]

Gel Extraction using MinElute Gel Extraction Kit:

6. Weigh a 2 mL micro tube with the gel slice
7. Add 2 volumes of Diffusion Buffer
8. Crash the gel slice using a 1 mL syringe plunger
9. Incubate at 50 ℃ for 30 min
10. Pass the gel slurry through Nanosep MF column by centrifugation at 15,000 rpm for 5 min
11. Measure volume of flow-through
12. Add 3 volumes of Buffer QG
13. Transfer 700 µL of the sample to an MinElute spin column placed in a 2 mL collection tube
14. Centrifuge at 20,000x*g*, room temp., 1 min, and then discard the flow-through
15. Transfer the remaining sample, repeat to centrifuge and discard
16. Add 500 µL of Buffer QG
17. Centrifuge at 20,000x*g*, room temp., 1 min, and then discard the flow-through

18. Add 750 µL of Buffer PE
19. Centrifuge at 20,000x*g*, room temp., 1 min, and then discard the flow-through
20. Re-centrifuge at 20,000x*g*, room temp., 1 min
21. Place the spin column in a new 1.5 mL collection tube
22. Add 20 µL DNase-free water directly to the center of the spin column membrane
23. Let stand for 1min
24. Centrifuge at 20,000x*g*, room temp., 1 min
25. Recover the flow-through (CLIP library)

Day 5

XIII. Quality & Quantity check

Measure peak size of CLIP library by TapeStation

Quantitate concentration of CLIP library by KAPA Library Quantification Kit

XIV. Sequencing

Dilute CLIP libraries to 2 nM each, mix in case of multiplex run

Sequence on MiSeq at 10-15 pM final concentration using Read 1 sequencing primer supplied in cartridge

Day X

S-I. 5'-adenylated 3'-linker preparation

<u>5' Adenylation:</u>

**<u>Reaction mix</u>**	
20 µM RL3	30 µL
10x 5' DNA Adenylation Reaction Buffer	12 µL
10 mM ATP	1.2 µL
Mth RNA ligase	6 µL
RNase-free water	70.8 µL

1. Prepare reaction mix in a 1.5 mL micro tube
2. Incubate at 65 ℃ for 1 hour
3. Incubate at 85 ℃ for 5 min to inactivate the enzyme
4. Let sit on ice

<u>RNA purification:</u>

5. Add 80 µL of RNase-free water
6. Add 200 µL of Acidic Phenol/Chloroform, mix by vortex mixer
7. Centrifuge at 20,000x*g*, room temp., 5 min
8. Transfer aqueous phase to a new 1.5 mL micro tube.
9. Add 20 µL of 3M NaOAc (pH5.5), 500 µL of 100% ethanol, 1.5 µL of GlycoBlue
10. Precipitate at -80 ℃ for 2 hours or overnight
11. Centrifuge the RNA at 20,000x*g* at 4 ℃ for 15 min
12. Wash 2 times with 1mL of cold 75% ethanol
13. Dry up the pellet, resuspend in 30 µL of RNase-free water
