# Supplementary figures and images for: Analysis of the nucleocytoplasmic shuttling RNA-binding protein HNRNPU using optimized HITS-CLIP method
Source: PLoS One. 2020 Apr 17;15(4):e0231450. doi: 10.1371/journal.pone.0231450 (PMC7164624; doi:10.1371/journal.pone.0231450)

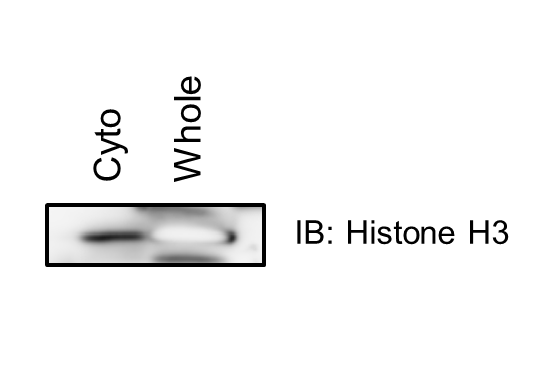

Supplement: S1 Fig — Western blotting of whole lysate (whole) and the cytoplasmic fraction (cyto) with Histone H3 (Related to Fig 6B). (TIF) [file pone.0231450.s006.tif]

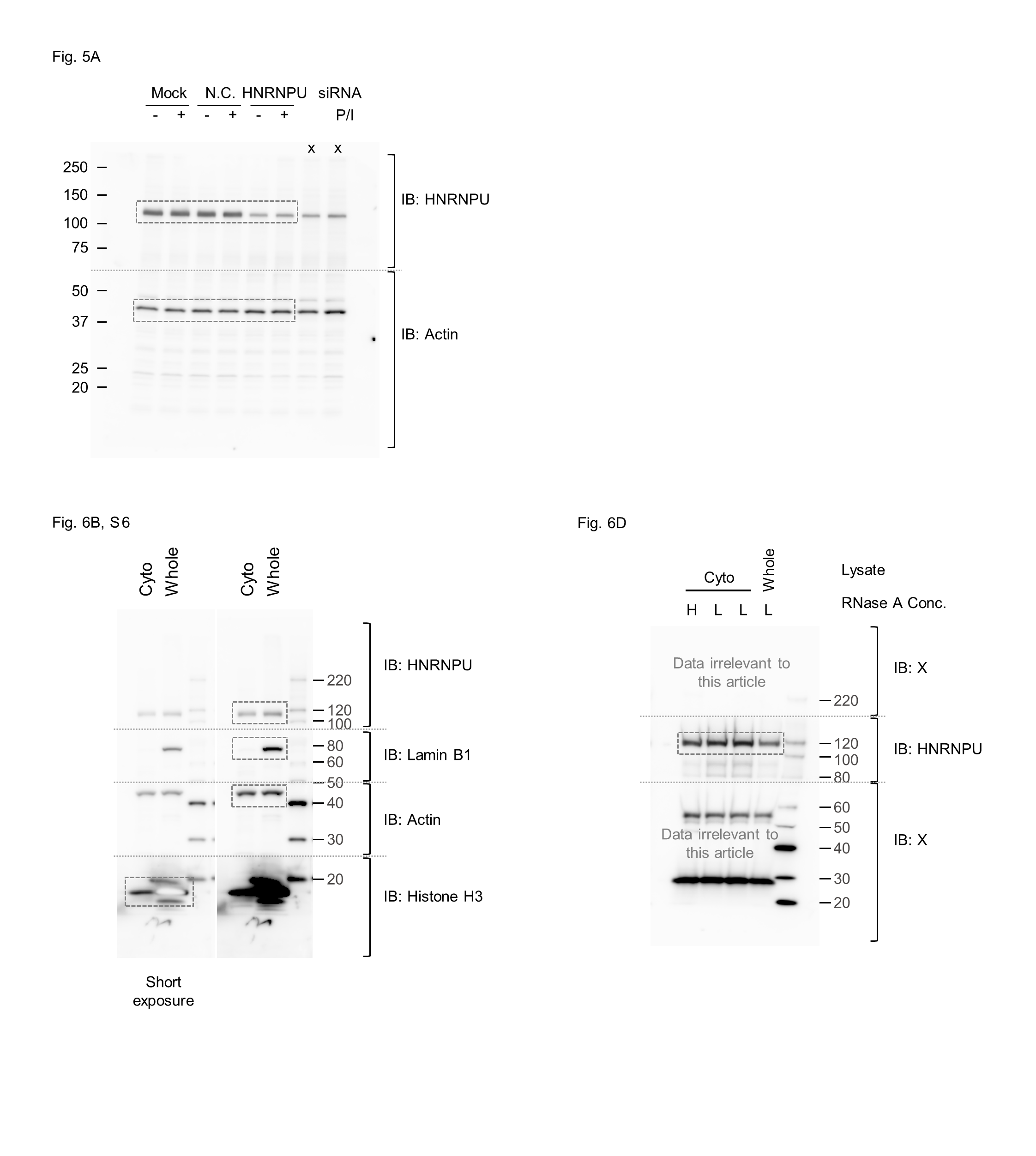

Supplement: S2 Fig — (TIF) [file pone.0231450.s007.TIF]
